# Integrative Analysis of HNF1B mRNA in Human Cancers Based on Data Mining

**DOI:** 10.7150/ijms.51213

**Published:** 2020-10-18

**Authors:** Chunhui Nie, Bei Wang, Baoquan Wang, Ning Lv, Enfan Zhang

**Affiliations:** 1 Hepatobiliary and Pancreatic Interventional Treatment Center, Division of Hepatobiliary and Pancreatic Surgery, The First Affiliated Hospital, Zhejiang University School of Medicine, Hangzhou 310003, Zhejiang Province, China; 2 Zhejiang Provincial Research Center for Diagnosis and Treatment of Hepatobiliary Diseases, Hangzhou 310003, Zhejiang Province, China; 3 Zhejiang Clinical Research Center of Hepatobiliary and Pancreatic Diseases, Hangzhou 310003, Zhejiang Province, China; 4 Department of Pharmacy, The First Affiliated Hospital, School of Medicine, Zhejiang University, Hangzhou, Zhejiang, China; 5 Bone Marrow Transplantation Center, Department of Hematology, The First Affiliated Hospital, School of Medicine, Zhejiang University, Hangzhou, Zhejiang, China

**Keywords:** HNF1B, cancer, immunotherapy, biomarker

## Abstract

Cancer incidence is rapidly growing, and cancer is the leading cause of death worldwide in the 21st century. Hepatocyte nuclear factor 1B (HNF1B) is a transcription factor that involves the growth and development of multiple organs. The aim of this study was to explore the significance of HNF1B in human cancer by an integrative analysis of online databases. The UALCAN database, cBio cancer genomics portal, Cancer Regulome tools, Kaplan-Meier plotter and Tumor IMmune Estimation Resource (TIMER) website were used to perform the corresponding analysis. The results showed that HNF1B is dysregulated in various cancers and associated with the differential overall survival of cancer patients. HNF1B showed many mutation forms and high mutation levels in different cancer types. In addition, we found that HNF1B interacted with different genes in multiple aspects. Moreover, HNF1B expression is associated with many immune cell infiltration levels and influences the prognostic prediction of immune cells in some kinds of cancers. In conclusion, HNF1B plays a significant role in cancer and may be a potential target for cancer immunotherapy.

## Introduction

Cancer incidence and mortality are rapidly rising worldwide^1^. Moreover, cancer is the leading cause of death and an important barrier to extending life expectancy in the 21st century. Despite the achievements in our understanding of the pathogenesis of cancer and the development of multidisciplinary therapies, the clinical outcomes for patients remain poor^2^-^4^. Therefore, there is an urgent need to discover potential biomarkers of cancer to predict the overall prognosis and improve the therapeutic management of patients.

Hepatocyte nuclear factor 1B (HNF1B) is located at chromosome 17q12, and it involves the growth and development of multiple organs, including liver, pancreas, kidney, etc^5^-^7^. During kidney development, HNF1B mainly regulates the growth and development of renal tubules^8^. HNF1B plays an indispensable role in all stages of pancreas development, including pancreatic endocrine cells and duct development^9^. Genes encoding HNF1B transcription factors are prone to various types of mutations, causing the occurrence and progression of various diseases, including diabetes, renal insufficiency, and various malignant tumors^10^-^12^.

In the present study, we evaluated expression and mutations of HNF1B in different types of cancer from The Cancer Genome Atlas (TCGA) database. The correlation between HNF1B expression and overall survival of cancer patients was further validated based on the Kaplan-Meier plotter. Moreover, we explored the association of HNF1B with immune cell infiltration levels and their prognostic values in cancer patients. The results of this study reveal the crucial role of HNF1B in cancer and an underlying mechanism between HNF1B and tumor-immune interactions.

## Material & Methods

### UALCAN database

The UALCAN database was employed to analyze the expression of HNF1B in tumors and normal tissues. This database includes RNA-seq and clinical information from the TCGA for 31 types of cancer^13^.

### Mutation analysis

The cBio cancer genomics portal was used to explore mutations and copy-number alterations of HNF1B in the TCGA pan-cancer studies. The cBio Cancer Genomics Portal provides rapid, intuitive, and high-quality access to the interactive analysis of multidimensional cancer genomics datasets^14^.

### Genome-wide association analysis

The Cancer Regulome tool was applied to explore the correlation between the expression levels of HNF1B and other genes in tumors. Spearman's correlation analysis based on the TCGA database was used to determine the correlation between two genes^15^.

### Kaplan-Meier plotter

The online Kaplan-Meier plotter database was used to explore the prognostic value of genes in cancer^16^. Kaplan-Meier survival plots were constructed to show the correlation between HNF1B expression and the overall survival of cancer patients. The statistical significance of the correlation was analyzed by the log-rank test.

### Immune-associated analysis

The Tumor IMmune Estimation Resource (TIMER) database was applied to estimate the correlations between HNF1B expression and immune cell infiltration levels^17^. The Estimating the Proportion of Immune and Cancer cells (EPIC) algorithm was also adopted in this study^18^. Moreover, we explored the outcome of cancer patients by comprehensively considering immune cell infiltration and HNF1B expression levels.

## Results

### Abnormal expression of HNF1B in cancer

**Figure 1:**
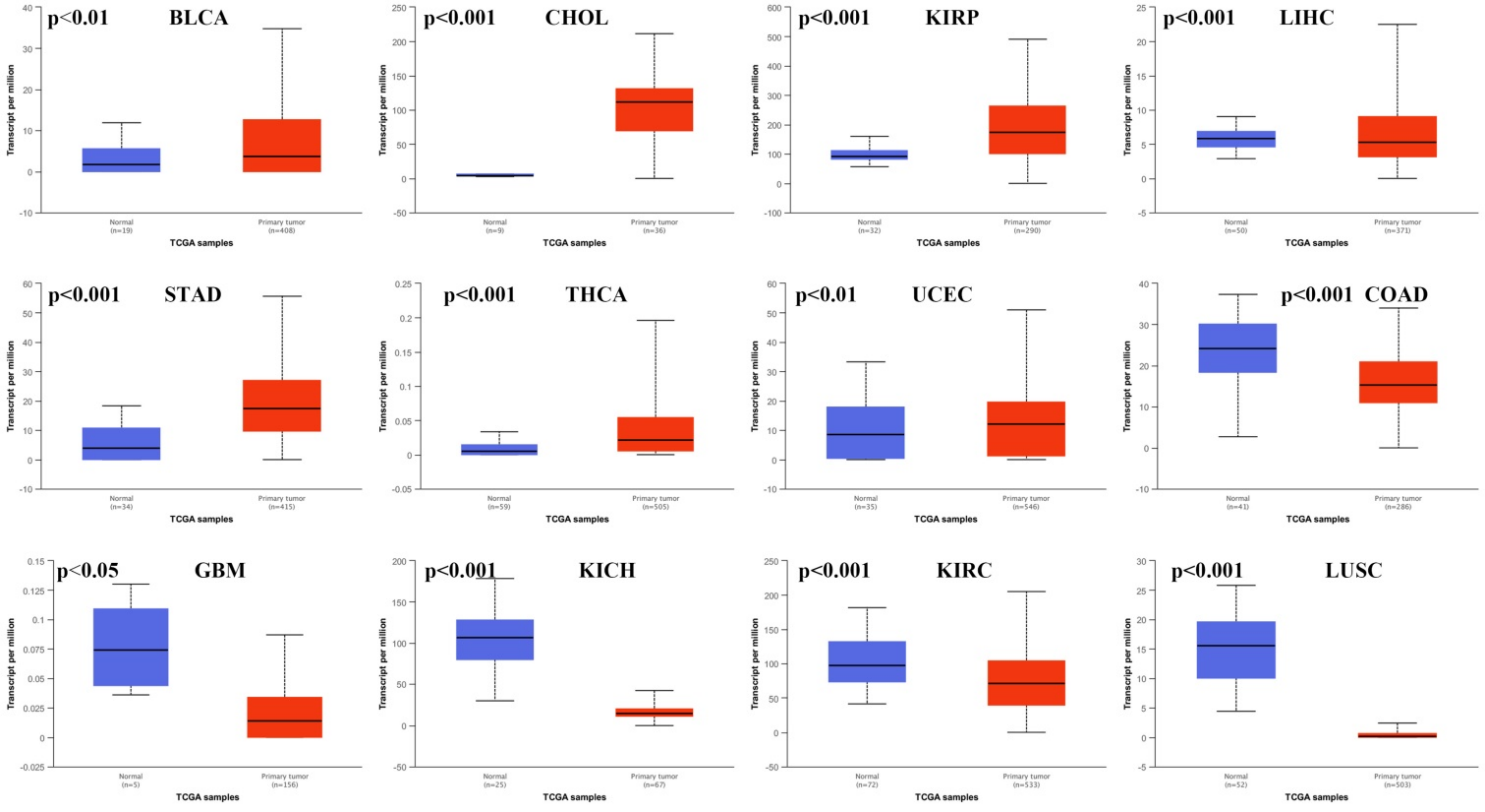
HNF1B mRNA expression was abnormal in various cancers based on UALCAN database analysis.

We used the UALCAN database to analyze the expression of HNF1B in different types of cancer. HNF1B levels in cancer tissues and in normal tissues were compared based on data from the TCGA. The results showed that HNF1B expression in various cancers was obviously different from that in normal tissues (Figure 1). The expression of HNF1B was upregulated in bladder urothelial carcinoma (BLCA), cholangiocarcinoma (CHOL), kidney renal papillary cell carcinoma (KIRP), liver hepatocellular carcinoma (LIHC), stomach adenocarcinoma (STAD), thyroid carcinoma (THCA), and uterine corpus endometrial carcinoma (UCEC), while the expression of HNF1B was downregulated in colon adenocarcinoma (COAD), glioblastoma multiforme (GBM), kidney chromophobe (KICH), kidney renal clear cell carcinoma (KIRC), and lung squamous cell carcinoma (LUSC). However, no significant differences were revealed in breast invasive carcinoma (BRCA), cervical squamous cell carcinoma (CESC), esophageal carcinoma (ESCA), head and neck squamous cell carcinoma (HNSC), lung adenocarcinoma (LUAD), pancreatic adenocarcinoma (PAAD), prostate adenocarcinoma (PRAD), or rectum adenocarcinoma (READ) (Supplementary Figure 1).

### HNF1B mutation in cancer

**Figure 2:**
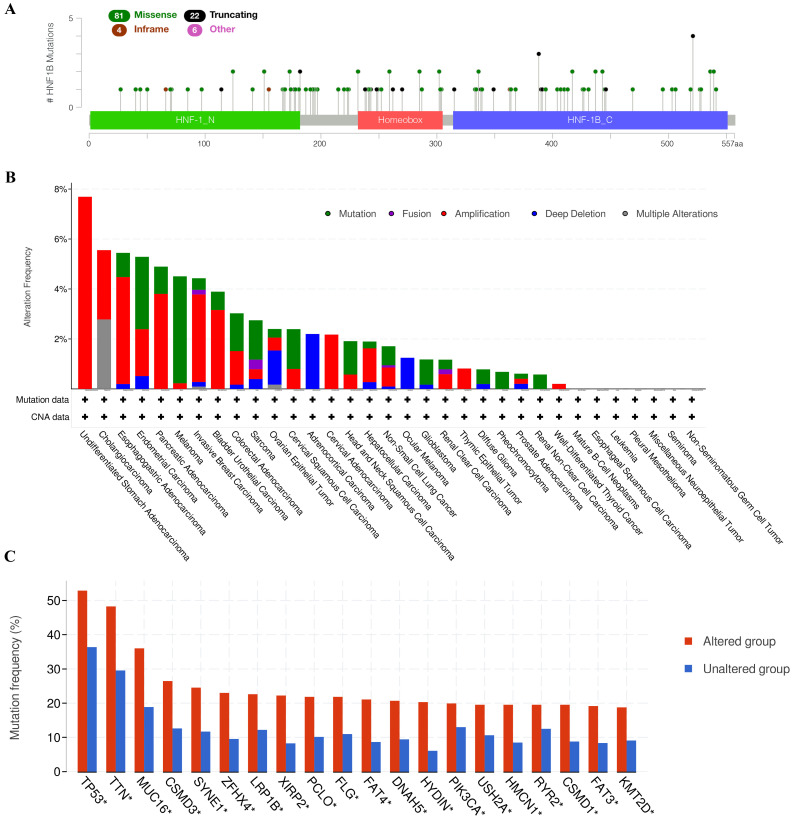
A. Mutation diagram of HNF1B in different cancer types across protein domains. B. HNF1B mutation and copy-number alterations in the TCGA pan-cancer studies. C. Genes with the top 20 highest frequency in any group (HNF1B-altered group and HNF1B-unaltered group). TP53-tumor protein p53; TTN-titin; MUC16-mucin 16; CSMD3-CUB and Sushi multiple domains 3; SYNE1-spectrin repeat containing nuclear envelope protein 1; ZFHX4 zinc finger homeobox 4; LRP1B-LDL receptor related protein 1B; XIRP2-xin actin binding repeat containing 2; PCLO-piccolo presynaptic cytomatrix protein; FLG-filaggrin; FAT4-FAT atypical cadherin 4; DNAH5-dynein axonemal heavy chain 5; HYDIN-HYDIN axonemal central pair apparatus protein; PIK3CA-phosphatidylinositol-4,5-bisphosphate 3-kinase catalytic subunit alpha; USH2A-usherin; HMCN1-hemicentin 1; RYR2-ryanodine receptor 2; CSMD1-CUB and Sushi multiple domains 1; FAT3-FAT atypical cadherin 3; KMT2D-lysine methyltransferase 2D.

We applied the cBio cancer genomics portal to explore the mutations of HNF1B in different cancers. As shown in Figure 2A, the mutation types of HNF1B included missense mutations, truncating mutations, in-frame mutations and other mutations. Moreover, HNF1B showed high mutation levels in Esophagogastric Adenocarcinoma, Endometrial Carcinoma, Pancreatic Adenocarcinoma, Melanoma, Invasive Breast Carcinoma, Bladder Urothelial Carcinoma, Colorectal Adenocarcinoma, Sarcoma, Ovarian Epithelial Tumor, Cervical Squamous Cell Carcinoma, Head and Neck Squamous Cell Carcinoma, Glioblastoma, Diffuse Glioma, Pheochromocytoma and Renal Non-Clear Cell Carcinoma. Additionally, cancer patients with HNF1B mutations are more susceptible to many other gene mutations, including TP53, TTN, MUC16, CSMD3, SYNE1, ZFHX4, LRP1B, XIRP2, PCLO, FLG, FAT4, DNAH5, HYDIN, PIK3CA, USH2A, HMCN1, RYR2, CSMD1, FAT3 and KMT2D (Figure 2C).

### Genome-wide association of HNF1B in cancer

**Figure 3:**
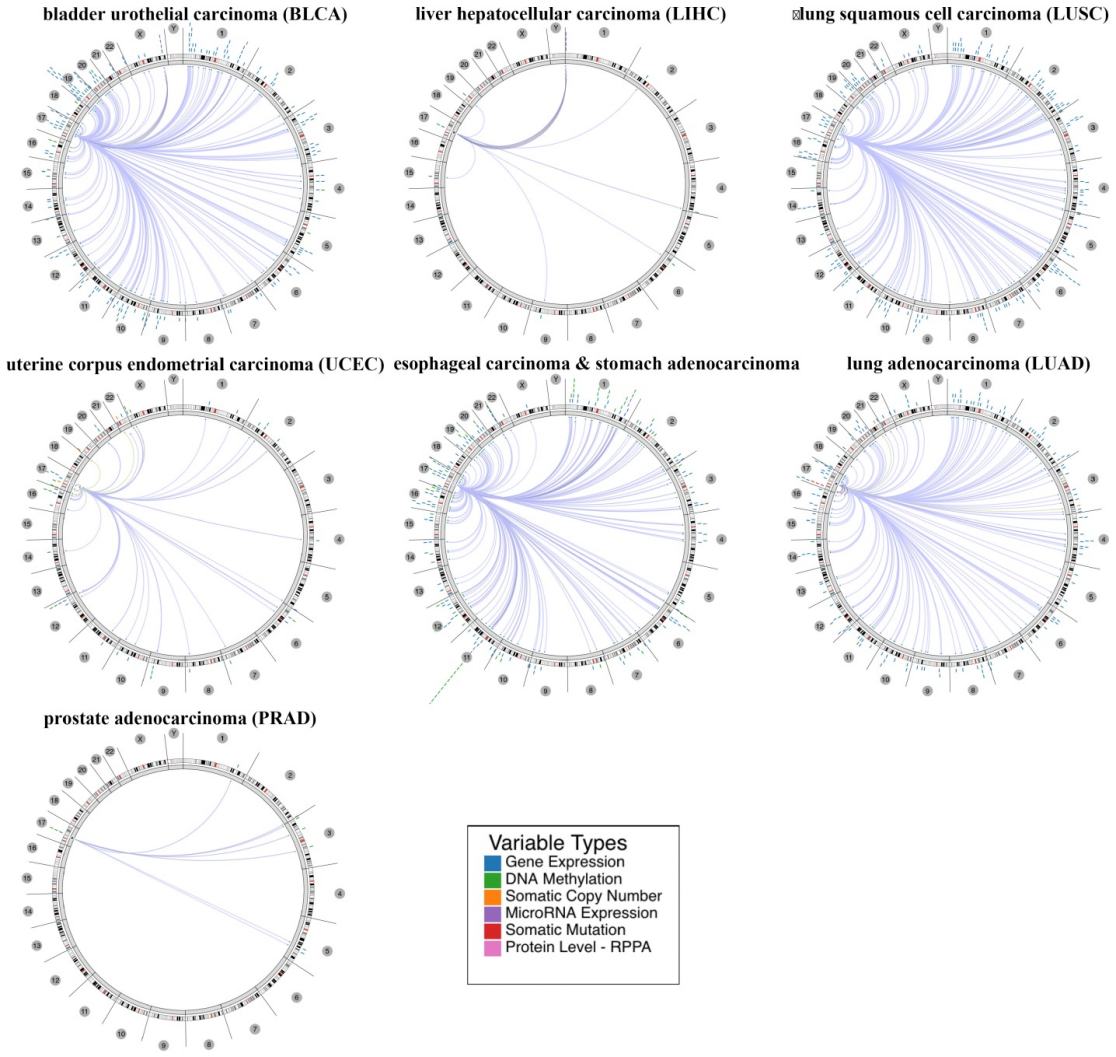
The correlation between HNF1B and other genes from the TCGA database was determined using the Cancer Regulome tool. Only genes with a correlation of Abs = 0.5 and p <0.01 are shown in the circus plots (200 max results).

Furthermore, we explored the relevant human genome location and the association between certain genes and HNF1B in human cancer based on a regulome explorer. Circus plots were drawn to reveal the correlation between HNF1B and other genes according to the association among genes, DNA methylation, somatic copy-number alteration, somatic mutation and protein level. As shown in Figure 3, HNF1B was associated with other genes that could be detected in bladder urothelial carcinoma, liver hepatocellular carcinoma, lung squamous cell carcinoma, uterine corpus endometrial carcinoma, esophageal carcinoma and stomach adenocarcinoma, lung adenocarcinoma and prostate adenocarcinoma.

### Survival analysis of HNF1B in cancer

**Figure 4:**
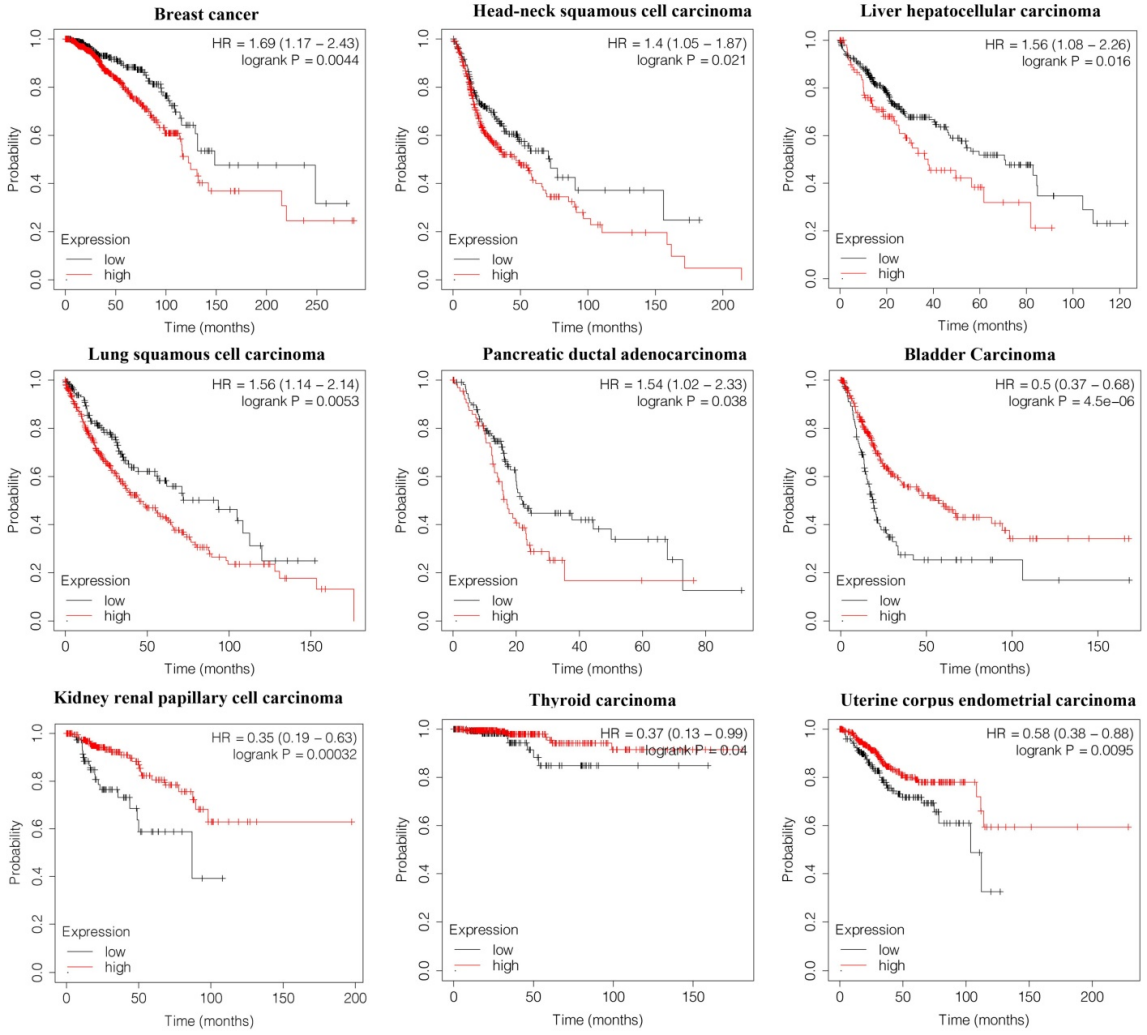
Correlation between HNF1B expression and the overall survival of cancer patients (Kaplan-Meier analysis).

To determine the potential value of HNF1B in predicting the overall survival of cancer patients, we constructed Kaplan-Meier survival curves. HNF1B was found to be negatively associated with overall survival in breast cancer (HR=1.69, p=0.0044), head-neck squamous cell carcinoma (HR=1.4, p=0.021), liver hepatocellular carcinoma (HR=1.56, p=0.016), lung squamous cell carcinoma (HR=1.56, p=0.0053), and pancreatic ductal adenocarcinoma (HR=1.54, p=0.038). However, higher levels of HNF1B indicated better overall survival in bladder carcinoma (HR=0.5, p=4.5e-06), kidney renal papillary cell carcinoma (HR=0.35, p=0.00032), (HR=0.37, p=0.04), and uterine corpus endometrial carcinoma (HR=0.58, p=0.0095) (Figure 4).

### HNF1B expression is correlated with immune infiltration levels in cancer

**Figure 5:**
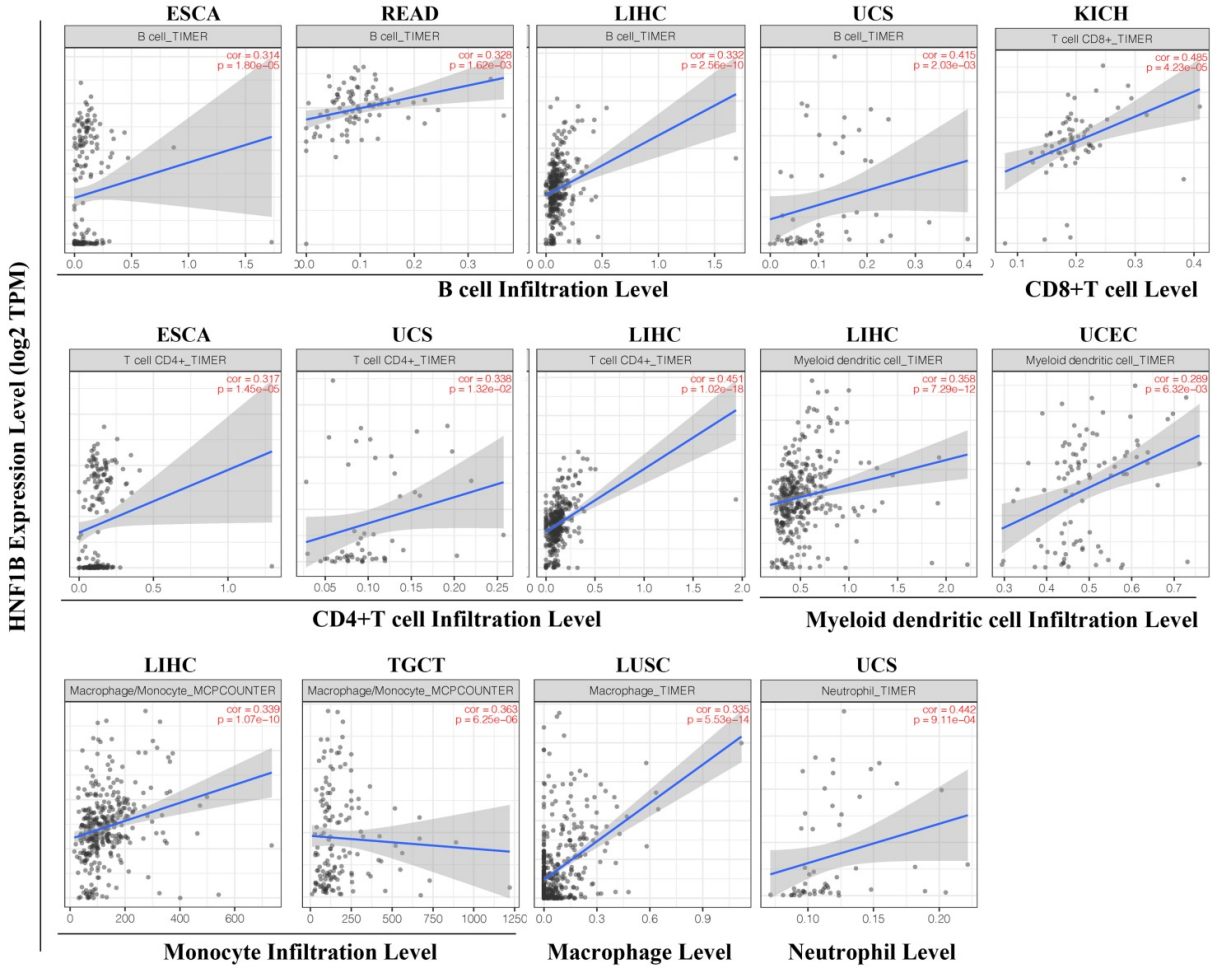
HNF1B expression is correlated with the immune infiltration levels in cancer (TIMER algorithm). ESCA: esophageal carcinoma, READ: rectum adenocarcinoma, LIHC: liver hepatocellular carcinoma, UCS: uterine carcinosarcoma, KICH: kidney chromophobe, UCEC: uterine corpus endometrial carcinoma, TGCTs: testicular germ cell tumors, LUSC: lung squamous cell carcinoma.

Based on the TCGA dataset, we evaluated the correlation of HNF1B with levels of immune cell infiltration in cancer from TIMER. As shown in Figure 5, HNF1B was significantly associated with B cells, CD8+ T cells, CD4+ T cells, myeloid dendritic cells (DCs), monocytes, macrophages and neutrophils in multiple tumors. A positive correlation between HNF1B expression and B-cell infiltration levels was found in esophageal carcinoma (ESCA, r = 0.314, p =1.80e-6), Rectum adenocarcinoma (READ, r=0.328, p=1.62e-03), liver hepatocellular carcinoma (LIHC, r= 0.332, p = 2.56e-10), Uterine Carcinosarcoma (UCS, r = 0.415, p =2.03e-03). A positive correlation between HNF1B expression and CD8+ T cell levels was found in Kidney Chromophobe (KICH, r=0.485, p=4.23e-05). Moreover, there was a strong positive correlation between HNF1B expression and CD4+ T cell levels in esophageal carcinoma (ESCA, r = 0.317, p =1.45e-5), Uterine Carcinosarcoma (UCS, r = 0.338, p =1.32e-02), liver hepatocellular carcinoma (LIHC, r= 0.451, p = 1.02e-18). In addition, the results also revealed a positive correlation between HNF1B expression and myeloid DC infiltration levels in liver hepatocellular carcinoma (LIHC, r= 0.358, p = 7.29e-12), Uterine Corpus Endometrial Carcinoma (UCEC, r=0.289, p=6.32e-03). The positive correlation between HNF1B expression and monocyte infiltration level was found in liver hepatocellular carcinoma (LIHC, r= 0.339, p = 1.07e-10) and Testicular Germ Cell Tumors (TGCT, r=0.363, p=6.25e-06). In Lung squamous cell carcinoma (LUSC), HNF1B was correlated with the macrophage infiltration (r=0.335, p=5.53e-14). In Uterine Carcinosarcoma (UCS), HNF1B was associated with the neutrophil infiltration (r=0.442, p=9.11e-04). Moreover, the EPIC algorithm analysis showed that HNF1B was significantly associated with NK cell and cancer-associated fibroblasts in cancer (Supplementary Figure 2).

### Comprehensive prognostic analysis of HNF1B and immune infiltration levels in tumors

**Figure 6:**
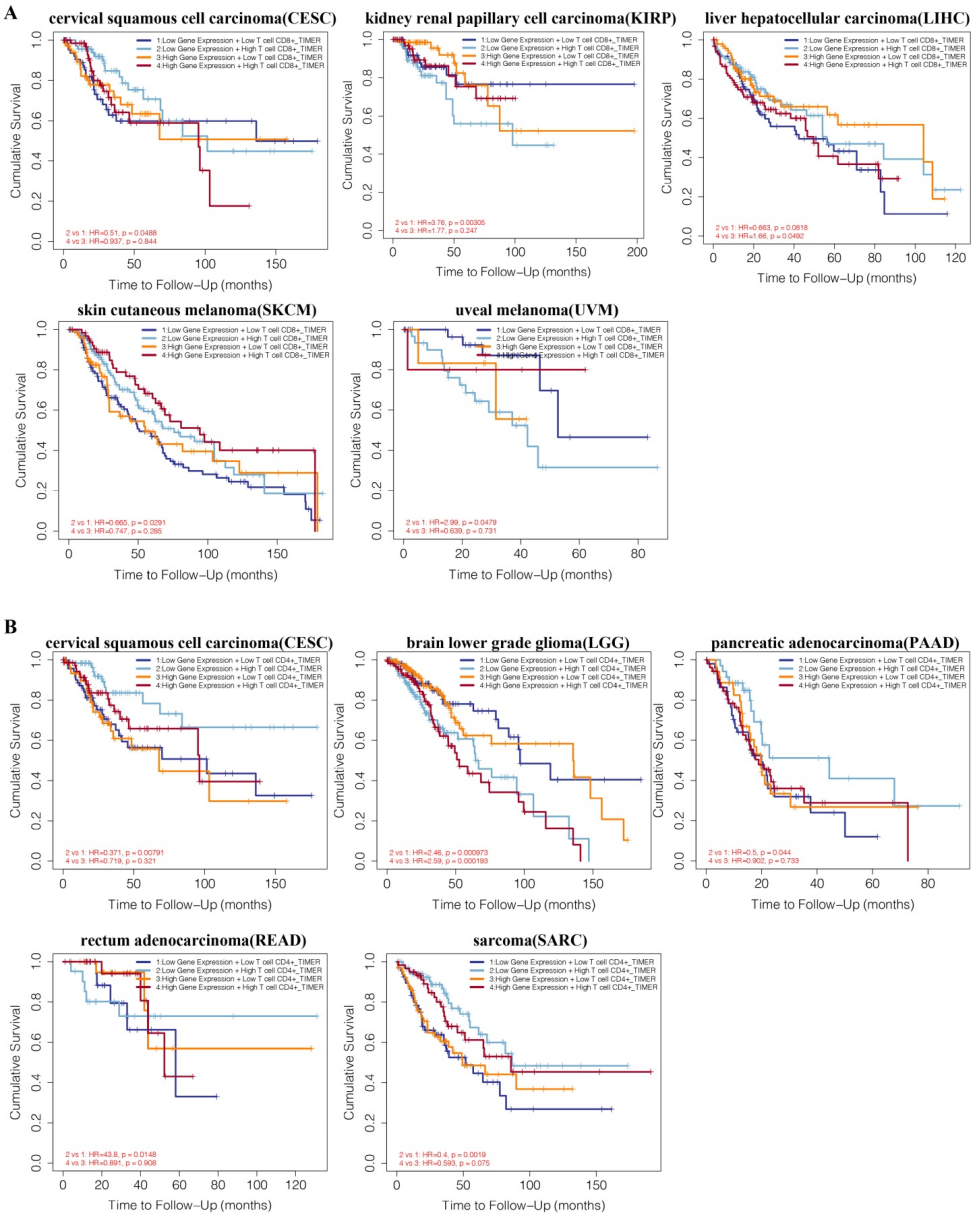
A. Comprehensive prognostic value of HNF1B expression and CD8+ T cell infiltration levels based on TIMER. B. Comprehensive prognostic value of HNF1B expression and CD4+ T cell infiltration levels based on TIMER.

We performed a prognosis analysis based on the HNF1B expression levels and immune cells in different tumors via TIMER. As shown in Figure 6A, CD8+ T cell levels were negatively associated with overall survival in the low HNF1B expression group of kidney renal papillary cell carcinoma (KIRP, HR=3.76, P=0.00305) and uveal melanoma (UVM, HR=2.99, P=0.0479). CD8+ T cells were found to be positively associated with overall survival in the low HNF1B expression group of cervical squamous cell carcinoma (CESC, HR=0.51, p=0.0488) and skin cutaneous melanoma (SKCM, HR=0.665, p=0.0291). In the low HNF1B expression group of liver hepatocellular carcinoma (LIHC), CD8+ T cells were revealed to be positively associated with overall survival. Additionally, the combination of HNF1B expression and CD4+ T cell infiltration levels showed prognostic value in cervical squamous cell carcinoma (CESC), brain lower grade glioma (LGG), pancreatic adenocarcinoma (PAAD), rectum adenocarcinoma (READ), sarcoma (SARC) (Figure 6B). In bladder urothelial carcinoma (BLCA), breast invasive carcinoma (BRCA), kidney renal clear cell carcinoma (KIRC), brain lower grade glioma (LGG) and mesothelioma (MESO), the expression level of HNF1B also influenced the prognostic value of B-cell infiltration levels (Supplementary Figure 3).

## Discussion

Hepatocyte nuclear factor (HNF) is a type of transcription factor that is distributed in the liver, pancreas, intestine, kidney and other organs, regulating the specific expression of a series of related genes^19^. HNF1, HNF3, HNF4 and HNF6 are its main subtypes, and these factors work together to coordinate and maintain the body's steady state. HNF1B is a member of the HNF family, participates in regulating the metabolism of lipids, carbohydrates and proteins, and plays an important role in regulating hepatocyte differentiation and liver development^20^. Recent studies have shown that HNF1B has a significant correlation with the occurrence and development of various tumors, such as prostate cancer and pancreatic cancer 21,22. In this study, we found that the expression of HNF1B was upregulated at different levels in BLCA, CHOL, KIRP, LIHC, STAD, THCA, and UCEC and downregulated in COAD, GBM, KICH, KIRC, and LUSC. Based on these results, HNF1B may be a good potential diagnostic biomarker for different cancers.

Consistent with the above results, the significance of HNF1B in hepatocellular carcinoma has been examined. Shim et al revealed that the expression of HNF1B predicted disease recurrence and hepatocellular carcinoma-specific death after liver transplantation in patients with hepatocellular carcinoma^23^. Another clinicopathological study showed that the HNF1B expression in hepatocellular carcinoma may be related with the change of phenotype on recurrence^24^. The authors also found that HNF1B expression was associated with poorer disease outcome, being an independent risk factor for both disease-free survival and overall survival. Moreover, it has been reported that the single nucleotide polymorphism (SNP) of HNF1B can affect the susceptibility of endometrial tumors. Spurdle et al. conducted gene sequencing studies on endometrial cancer patients and control groups and found that HNF1B gene SNP (rs4430796, G→A) can reduce the incidence of endometrial cancer^25^. Meta-analysis of large samples also confirmed this result^26^. At the same time, the HNF1B gene can independently affect the prognosis of endometrial tumors^27^.

The HNF1B gene is located on chromosome 17q12 and contains 9 exons, which have 3 different functional domains^28^. Most HNF1B mutations are clustered in the first 4 exons of the gene. HNF1B has frequent spontaneous neonatal mutations, and the spontaneous deletion rate is up to 50%. A total of 106 HNF1B gene mutations, including gene deletion (34%), missense mutation (31%), frameshift deletion or insertion mutation (15%), nonsense mutation (11%) and splicing point mutation (8%) have been reported^29^,^30^. According to these results, our study found that HNF1B mutations occurred widely in human cancers and that the most common type is missense mutations. Additionally, patients with HNF1B mutations are more prone to mutations in other genes, such as TP53, TTN and MUC16. Survival analysis revealed that HNF1B expression indicated distinct overall survival rates of cancer patients. The distinct prognostic value of HNF1B may be caused by the different mutations and interactions between HNF1B and other genes in different types of cancers.

Immune cells that infiltrate tumors play crucial roles in tumor development^31^. Evading immune destruction is one of the hallmarks of cancer^32^. The role of the immune system in cancer oncogenesis is increasingly being investigated, and immunotherapy has become a powerful clinical strategy for treating cancer^33^,^34^. Our study showed that HNF1B expression is associated with diverse immune cell infiltration levels in different cancers. The results demonstrated a strong positive correlation between HNF1B expression and the infiltration levels of B cells, CD8+ T cells, CD4+ T cells, macrophages, neutrophils and DCs in cancer. Additionally, we divided patients into a low HNF1B expression group and a high HNF1B expression group, and CD8+ T cell, CD4+ T cell and B-cell levels were distinctly associated with overall survival of cancer patients in the different groups. The association of HNF1B and immune cells has rarely been studied^35^ and needs to be further investigated. HNF1B expression may be a considerable factor for patients who are going to adopt immunotherapy.

In conclusion, our study found that HNF1B mRNA is dysregulated in various cancers and that the differential expression of HNF1B indicates different prognoses. HNF1B mutations are widely observed in tumors and interact with different genes in different cancer types, which may be the cause of the distinct prognostic values in cancers. Moreover, HNF1B is associated with the level and prognosis prediction of immune cells in some kinds of cancers.

## Supplementary Material

Supplementary figures.

## Abbreviation

HNF1B: Hepatocyte nuclear factor 1B
TIMER: Tumor IMmune Estimation Resource
TCGA: The Cancer Genome Atlas
EPIC: Estimating the Proportion of Immune and Cancer cells
BLCA: bladder urothelial carcinoma
CHOL: cholangiocarcinoma
KIRP: kidney renal papillary cell carcinoma
LIHC: liver hepatocellular carcinoma
STAD: stomach adenocarcinoma
THCA: thyroid carcinoma
UCEC: uterine corpus endometrial carcinoma
COAD: colon adenocarcinoma
GBM: glioblastoma multiforme
KICH: kidney chromophobe
KIRC: kidney renal clear cell carcinoma
LUSC: lung squamous cell carcinoma
BRCA: breast invasive carcinoma
CESC: cervical squamous cell carcinoma
ESCA: esophageal carcinoma
HNSC: head and neck squamous cell carcinoma
LUAD: lung adenocarcinoma
PAAD: pancreatic adenocarcinoma
PRAD: prostate adenocarcinoma
READ: rectum adenocarcinoma
UCS: Uterine Carcinosarcoma
UVM: uveal melanoma
SKCM: skin cutaneous melanoma
DCs: dendritic cells
LGG: brain lower grade glioma
SARC: sarcoma
MESO: mesothelioma
