# Supplementary material for: Integrative Analysis of HNF1B mRNA in Human Cancers Based on Data Mining
Source: Int J Med Sci. 2020 Oct 18;17(18):2895–904. doi: 10.7150/ijms.51213 (PMC7646120; doi:10.7150/ijms.51213)
Supplement: Supplementary file 1 — Supplementary figures. [file ijmsv17p2895s1.pdf]

Supplementary Figures

Supplementary Figure 1

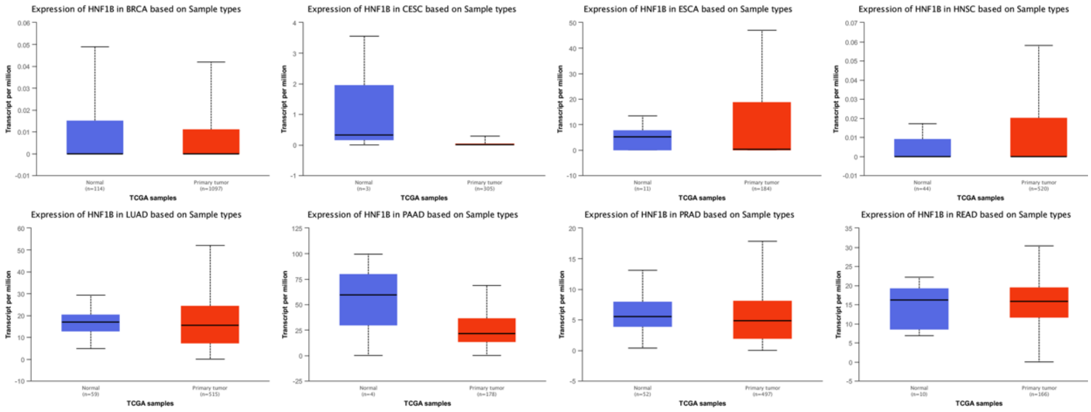

Supplementary Figure 1. HNF1B expression in tumor tissues and normal tissues (without significant differences).

Supplementary Figure 2

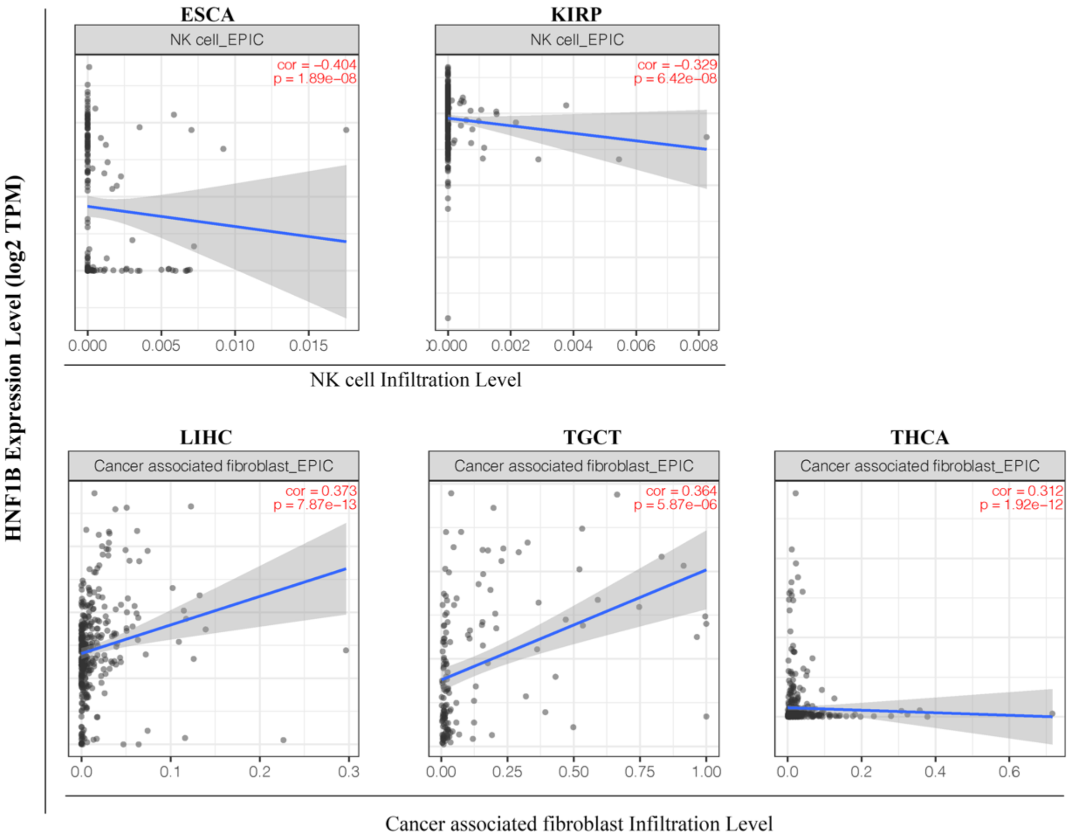

Supplementary Figure 2. HNF1B expression is correlated with NK cells and cancer-associated fibroblast infiltration levels in cancer (EPIC algorithm). ESCA: esophageal carcinoma, KIRP: kidney renal clear cell carcinoma, LIHC: liver hepatocellular carcinoma, TGCTs: testicular germ cell tumors, THCA: thyroid carcinoma.

Supplementary Figure 3

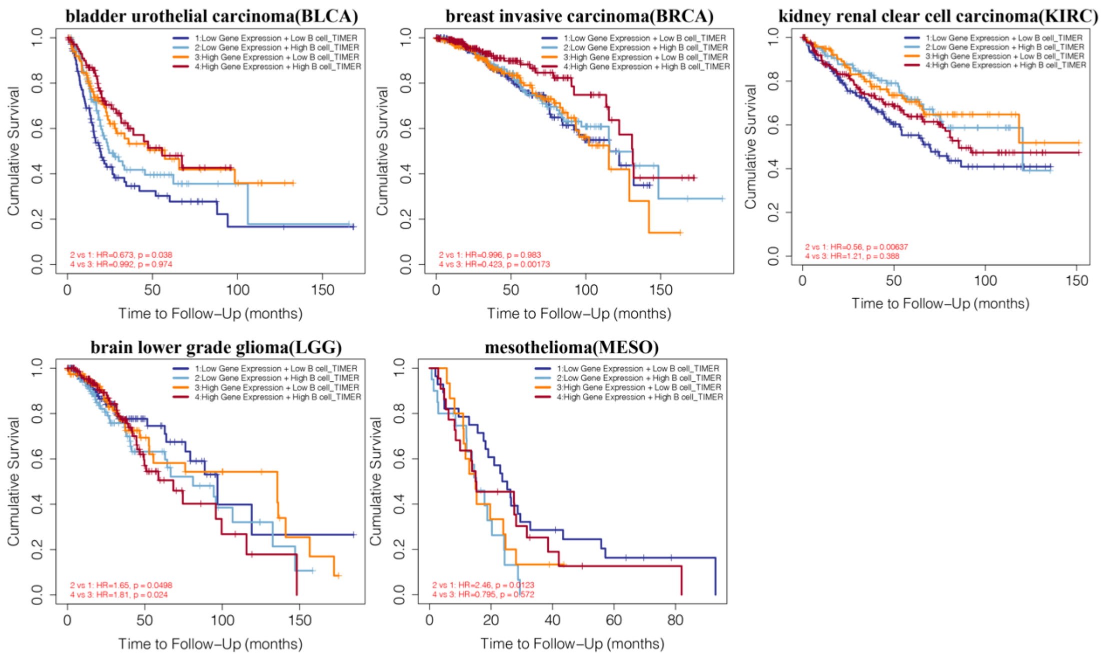

Supplementary Figure 3: The prognostic value of HNF1B expression and B-cell infiltration levels based on TIMER.
